# Drug, Herb, and Dietary Supplement Hepatotoxicity

**DOI:** 10.3390/ijms17091488

**Published:** 2016-09-06

**Authors:** Rolf Teschke, Raúl J. Andrade

**Affiliations:** 1 Department of Internal Medicine II, Division of Gastroenterology and Hepatology, Klinikum Hanau, Academic Teaching Hospital of the Medical Faculty, Goethe University Frankfurt/Main, Frankfurt am Main, D-63450 Hanau, Germany; 2 Liver Unit Gastroenterology Service, Institute for Biomedical Research of Malaga (IBIMA), Virgen de la Victoria University Hospital and School of Medicine, 29010 Malaga, Spain; 3 Centro de Investigación Biomédica en Red de Enfermedades Digestivas y Hepáticas (CIBERehd), 28029 Madrid, Spain

The past decade has witnessed drugs, herbs, and dietary supplements share the common feature of potential liver injury in a few susceptible individuals. Most of these hepatotoxic reactions are idiosyncratic and hence difficult to predict. Treatment commonly consists of the cessation of the incriminated product, but causality attribution may be cumbersome due to a lack of valid diagnostic biomarkers. To aid diagnosis in hepatotoxicity cases, diagnostic tools are available, such as RUCAM (Roussel Uclaf Causality Assessment Method), which considers hepatotoxicity specific diagnostic key items with individual scorings, and the method of DILIN (Drug-Induced Liver Injury Network), which is based on expert opinion. Whereas hepatotoxicity by synthetic drugs is fairly well recognized in clinical practice and trials, the diagnosis of liver injury by herbs, herbal drugs, and dietary supplements is commonly challenging. Liver injury by these three compound groups presents similar clinical features; as patients with liver injury often have used several products, this co-administration complicates attributing liver injury to a single product as cause.

Case characterization of drug-induced liver injury (DILI) as well as herb-induced liver injury (HILI) requires liver injury cases with established causality for any of the consumed products. Unfortunately, many published case reports of liver injury lack such robust causality assessment and provide vague results, whereas the majority of case series present cases with well-established causality, which can be used to elucidate issues of clinical relevance of such disease phenotypes and genetic or environmental risk factors including lifestyle factors and questions of drug dosing. There is also a need of additional research to find diagnostic biomarkers which can help to establish the diagnosis of idiosyncratic liver injury, whereas such biomarkers are already available for a few drug or herbal products such as acetaminophen or unsaturated pyrrolizidine alkaloids as ingredients of selected herbs.

The aim of this special issue is to provide a broad overview on these hepatotoxicity entities with their challenges and highlights. We therefore have asked experts in the field to contribute their views on this emerging and exciting topic and have welcomed other experts as contributors. Since various topics are still controversial and disputed, we expect and appreciate lively discussions in addition to well settled issues that are relevant to clinical settings and require balanced statements.

Hallmarks of clinical liver injury will be presented in the Special Issue “Drug, Herb, and Dietary Supplement Hepatotoxicity” published in the *International Journal of Molecular Sciences* [1] to address the progress and current standing in the vast field of liver injury. A total of 12 reviews and other articles have been published in this Special Issue as detailed in Table 1.

The Special Issue opens with the review of Danan et al. [2] on RUCAM, the Roussel Uclaf Causality Assessment Method, which is a well-established tool in common use to quantitatively assess causality in cases of suspected drug-induced liver injury (DILI) and herb-induced liver injury (HILI). RUCAM represents a structured, standardized, validated, and hepatotoxicity specific diagnostic approach that attributes scores to individual key items of hepatotoxicity, providing final quantitative gradings of causality for each suspected drug or herb in a case report. RUCAM thereby circumvents experts’ and clinicians’ problems with unstructured evaluations lacking defined and scored items that commonly results in debated causality assignments. In 1993, experts from Europe and the United States had established in consensus meetings the first criteria of RUCAM to meet the requirements of clinicians and practitioners in care for their patients with suspected DILI and HILI. RUCAM was completed by additional criteria and validated also by positive reexposure tests, assisting to establish the timely diagnosis with a high degree of certainty. In many countries and for more than two decades, physicians, regulatory agencies, case report authors, and pharmaceutical companies successfully applied RUCAM for suspected DILI and HILI. Their practical experience, emerging new data of DILI and HILI characteristics, and a few ambiguous questions in domains such as alcohol use and exclusions of non-drug causes like hepatitis E virus (HEV) led to the present update of RUCAM. The aim was to reduce interobserver and intraobserver variability, to provide accurately defined, scored objective key elements, and to simplify the handling of the items. The authors now present the update of the well-accepted original RUCAM scale and recommend its use for clinical and regulatory publications and expert purposes to validly establish causality in cases of suspected DILI and HILI, facilitating a straight-forward application and an internationally harmonized approach of causality assessment as a common basic tool.

In the second review, Hayashi [3] discusses details of the DILIN expert opinion process, its strengths and weaknesses, psychometric performance, and the future. In 2004, the U.S. Drug-Induced Liver Injury Network (DILIN) was created under the auspices of the U.S. National Institute of Diabetes and Digestive and Kidney Diseases with the aims of establishing a large registry of cases for clinical, epidemiological, and mechanistic study. From inception, the DILIN has used an expert opinion process that incorporates consensus amongst three different DILIN hepatologists assigned to each case, yet it is an imperfect standard and has no translation into daily clinical practice. By definition, such experts’ approach is not validated by a gold standard and lacks transparency as to how final causality assessments were reached, which excludes any re-assessment and critical discussion. Although some of the hepatotoxicity criteria of RUCAM were seemingly already used in the DILIN method, a scoring of key items was not considered for a causality assessment. Based on US experts, its use is confined to the Continental US, excluding for instance Hawaii. It is stated that liver biopsy is not required, but every effort is made to obtain liver tissue blocks and slides of any biopsy. How and whether to incorporate the DILIN expert reading of liver biopsies is being considered, but incorporating such data into causality assessments is seen as a challenge. Reported also are lengthy and lively conversations that often occur in the DILIN consensus process, as reviewers point out overlooked data, weaknesses in reasoning, or data from new publications. Other weaknesses and limitations of the DILIN process are openly addressed. These include the statement that the DILIN process is cumbersome, time-consuming, and costly, and approaches to improve the process are continually sought. To overcome these obstacles, others could argue why a transatlantic approach incorporating RUCAM in the DILIN process is not used.

In his critical analysis of published DILI cases, Björnsson [4] discusses controversies as to what extent published reports of hepatotoxic drugs are valid with respect to their proposed hepatotoxic risk. Referring to the LiverTox^®^ website, this was an attempt to provide up-to-date, accurate, and easily accessible information on the diagnosis, causes, frequency, and patterns of liver injury attributable to both prescription and nonprescription medications. Although in LiverTox^®^ a thorough literature search has been undertaken and is provided, no attempt has been made to judge the data quality or causality grading of the published reports. Indeed, in a recent study of drugs with rather few reports, RUCAM was used to verify causality with the result that many of the published reports did not stand up to critical review, and currently there is no convincing evidence for some drugs with reported hepatotoxicity to be in fact hepatotoxic. The conclusion may be reached that RUCAM use is helpful to attribute firm causality levels in some drugs or dismiss causality in other medications. An additional analysis of the hepatotoxicity of drugs found in LiverTox revealed that fewer drugs than expected had documented hepatotoxicity. Among 671 drugs available in LiverTox, 318 drugs (47%) and thereby almost half had no published convincing case reports of hepatotoxicity. Herbal and dietary supplements listed in LiverTox were explicitly not included in the analysis, but results likely will be again poor and disappointing. Other drug groups had case reports only with a possible likelihood score according to RUCAM. There is also the note that the poorly documented exclusion of competing causes as well as other concomitant drugs made a causality assessment difficult and problematic in many reports. Provided is also a list of the most common implicated drugs, very useful information for clinicians in care of patients with DILI. Specifically mentioned here are azathioprine and infliximab.

Ortega-Alonso et al. [5] cover in their review the broad field of DILI case characterization with focus on clinical features, host factors, and drug chemical hazards. Based on carefully quoted references, they discuss data indicating that (1) an association exists of serious hepatic events such as liver failure, liver related liver transplantation, and liver-related death with a higher dosage of drugs (≥50 mg per day); (2) drugs with a recommended daily dose of ≥50 mg are responsible for at least 77% of all DILI cases; (3) drugs that undergo extensive hepatic metabolism (>50%) are also associated with a greater risk of DILI; and finally (4) high drug lipophilicity and bile salt export pump inhibitory properties, particularly when the drug has also mitochondrial effects that are considered risk factors, although the specificity of these chemical hazards in predicting DILI risk is low. Another highlight was the question as to whether pre-existing liver disease could be a risk factor of DILI, which is still a disputed issue. Under discussion are drugs such as antiviral and antiretroviral medications, antituberculous drugs, and methotrexate and chronic hepatits B and C, as well as patients with HIV/AIDS infection. Besides, individuals with pre-existing liver disease, which mainly included hepatitis C and NAFLD, were found to have higher mortality from DILI (16% versus 5.2%), but liver-related mortality was not increased in patients with underlying liver disease, indicating that other comorbidities such as diabetes or metabolic syndrome may have contributed to the excess of mortality. The rarity of idiosyncratic DILI upon exposure to the otherwise safe drugs for most individuals suggests that genetic variants make some individuals susceptible for liver injury. For some drugs such as amoxicillin-clavulanate, fenofibrate, flucloxacillin, lapatinib, lumiracoxib, ximegalatran, ticlopidine, and terbinafine, specific human leucocyte antigen (HLA) alleles have been found as risk factors of DILI.

DILI registries are of increased importance for expansion of clinical and basic research studying and further identifying DILI case phenotypes. In this connection, Bessone et al. [6] review the Latin American (LA) DILI Registry experience, which is described as a successful ongoing collaborative strategic initiative with the Spanish DILI Registry, which provided overall support based on long-standing expertise, initial concepts, and operational structures including a RUCAM-based causality assessment procedure, to build up the new LA DILI Registry. The main goal of the creation of this DILI registry in LA was the prospective and standardized identification of the different manifestations that DILI disease has in this region, so as to obtain highly valuable information of patient characteristics, most frequently involved drugs or herbal supplements, phenotypic presentations, and outcomes. Among the major objectives is also the collection of data to gain a better understanding of the environmental and host factors in DILI in comparison with figures reported by other registries. The LA DILI Registry collected carefully the DILI cases and excluded 51 cases. The causes of exclusion were as follows: not meeting DILI criteria; lack of viral serology and imaging data; uncertainty of treatment duration; and hepatic disease of other etiology including autoimmune hepatitis, biliary obstruction, ischemic hepatitis, hepatitis A, C, and E, and alcoholic hepatitis. All suspected DILI cases were submitted to RUCAM to assign the score of probability. RUCAM-based causality was analyzed in 197 cases, and causality grading was highly probable (9%), probably (67%), and possible (24%). The main 10 drugs and leading the LA DILI Registry were as follows: amoxicillin-clavulanate (*n* = 20), diclofenac (*n* = 13), nimesulide (*n* = 11), nitrofurantoin (*n* = 11), cypropterone acetate (*n* = 9), ibuprofen (*n* = 7), RIP + PZA + INH (*n* = 7), carbamazepine (*n* = 5), phenytoin (*n* = 4), and thiamazole (*n* = 4). Overall, this review hopefully will encourage and assist the introduction of other DILI registries in more countries.

In the next section of the Special Issue that is devoted to herb-induced liver injury (HILI), Frenzel et al. [7] describe the clinical case phenotypes and provide a listing compilation of herbs, which were incriminated as causative products with suspected hepatotoxic potential as published in various case reports or case series. Among these herbs are those used in Western herbal medicine as well as herbal TCM (Traditional Chinese Medicine). Most of these herbs are well tolerated by the majority of individuals, but liver injury may rarely occur in susceptible individuals with the risk of acute liver failure and requirement of liver transplantation. Phytochemicals are foreign products to the body and need metabolic degradation to be eliminated. During this process, hepatotoxic metabolites may be generated causing liver injury in susceptible patients. There is uncertainty as to whether risk factors such as high lipophilicity or high daily and cumulative doses play a pathogenetic role for HILI in analogy to DILI. Some herbs containing unsaturated pyrrolizidine alkaloids cause hepatic sinusoidal obstruction syndrome (HSOS), recognized by pyrrole-protein adducts as specific diagnostic biomarkers in the blood. In other cases, HILI is a diagnosis of exclusion, because clinical features of HILI are not specific as they are also found in many other liver diseases unrelated to herbal use. To establish HILI as the cause of liver damage, RUCAM is the preferred causality assessment tool; if available, unintentional positive reexposure is of additional diagnostic value. With these approaches, causality was established for some herbs. Problems may emerge when alternative causes were not carefully excluded and the correct therapy is withheld. Clearly, herbal use has to be stopped if HILI is suspected. Finally, all herbs and herbal dietary supplements used as medicine should be classified as herbal drugs with strict regulatory surveillance, ascertaining an appropriate risk benefit balance.

Other aspects of herbal medicine and related hepatotoxicity are outlined by Valdivia-Correra et al. [8] in their critical review referring to HILI in Mexico, where herbal medicine is commonly used, but side effects including liver injury are not yet thoroughly enough investigated. During the sixteenth century, numerous documents highlight the importance of herbal medicine in Mexico’s history. However, only during the 1990s, ethnobotanical research began to develop, combining ethnopharmacology and ethnobotany as the principal avenues of study. In Mexico, the distribution of herbal medicine correlates well with the regions of inhabited by indigenous communities. The four most common used herbs as medicines are *Cestrum nocturnum*, *Psidium guajava*, *Aritolochia odoratissima*, and *Zingiber officinale*. Dermatological and gastrointestinal disorders were the most frequently cited medical problems for which these were used. In the Digital Library of Traditional Mexican Medicine presented by the National Autonomous University of Mexico, seven herbal products with warnings about hepatotoxicity are provided: *Scoparia dulcis* L. (maidenhair), *Citrus aurantium* L. (citrus orange), *Prunus persica* L. (peach), *Rosmarinus officinalis* L. (rosemary), *Equisetum hyemale* L. (horse tail), *Tilia mexicana Schlechtendal* (tilia), and *Morus alba* L. (white mulberry). Most of these herbal products are used daily by the Mexican population. Potential hepatotoxicity was also discussed in various publications for other herbs consumed in Mexico including peppermint (*Mentha piperita*), chaparral (*Larrea divaricata*), dandlion (*Taraxacum officinale*), mullein (*Verbascum densiflorum*), chamomile (*Matricaria recutita*), nettle or stinging nettle (*Urtica dioica*), passionflower (*Passiflora incarnata*), linden flower (*Tilia europea*), and aloe (*Aloe vera*). However, little is known as to whether any causality assessment method was used to verify a causal relationship; reported hepatotoxicity is therefore vague. The authors also conclude that present knowledge of the therapeutic benefits and risks of some herbal medicines used in Mexico is still limited, and efforts to elucidate them should be intensified. 

Cases of herb-induced liver injury gathered in the Berlin Case-Control Surveillance Study are presented by Douros et al. [9] and critically evaluated. This is one of the few reports of HILI in Germany, showing that its prevalence is surprisingly low, although this country is known for its preference of herbal use. The study was initiated in 2000 to investigate the serious toxicity of drugs including herbal medicines. Potential cases of liver injury were ascertained in more than 180 departments of all 51 Berlin hospitals from October 2002 to December 2011. Drug or herb intake was assessed through a standardized interview, and causality for drugs or herbs was evaluated using RUCAM. Among all 198 hepatotoxicity cases included in the study, 10 were HILI cases with a RUCAM-based probable causality for Ayurvedic herb (*n* = 1), and possible causality for *Valeriana* (*n* = 5), *Mentha piperita*, *Pelargonium sidoides*, *Hypericum perforatum*, and *Eucalyptus globulus* with 1 case each. Mean age was 56.4 ± 9.7 years, and the predominant pattern of liver injury was hepatocellular. No cases of acute liver failure or death were observed. However, critical was the note that a possible causality does not prove clinical significance. The authors correctly address other limitations of this study. RUCAM-based scores are low in most of the reported HILI cases, suggestive of a non-herb etiology. Additionally, since HILI is a diagnosis of exclusion and relatively common hepatitis causes such as infections by cytomegalovirus, Epstein-Barr virus, hepatitis E virus (HEV), herpes simplex virus, and varicella zoster virus were rarely excluded in the study patients. An infectious etiology cannot be ruled out for every case. The authors remind us that this is particularly important for HEV, since recent publications suggested that, although accounting for some cases of suspected drug-induced liver injury, serologic testing excluding a respective HEV infection was not regularly performed.

In their review on “the 3Ks”—kava, kratom, and khat—Pantano et al. [10] discuss the uncertainties and controversies around the hepatotoxic potential of these three herbs and the possible pathogenetic mechanisms of their liver injury. They confirm that causality has been established in some cases of kava hepatotoxicity using RUCAM, but such a method assessing causality evaluation has not yet been applied in suspected cases of kratom and khat. Kava has its origin in the South Pacific Islands, where it is consumed for recreational purposes; in Western countries, kava was used as an efficient anxiolytic herb before it was withdrawn from the markets of many countries including Germany due to suspected liver injury. In Germany, kava had the status of a regulatory approved herbal drug with regulatory surveillance. As RUCAM-based occurrence of liver toxicity due to kava products seemed to be extremely rare, the regulatory ban imposed on the kava drug in Germany in 1992 was overturned in 2014 after decisions of administrative German Courts. In the few liver injury cases due to kava, problems were recognized that included, among others, a lack of compliance to recommended dose with overdosing, and co-ingestion of herbal supplements or other drugs. There were also issues of non-standardization of the kava products. Of note, kava was continuously available in the US with a cautionary statement and it is well accepted in Australia, currently with clinical trials for anxiety disorders, using an aqueous extract of the noble kava variety Borogu. The authors also provide information on kratom, which is known for its antinociceptive, opioid-like effects that are 13-fold stronger than morphine. Countries or areas of its origin include Malaysia, Bali, Thailand, Borneo, New Guinea, Java, Sumatra, and others. Grown in the East and the Horn of Africa as well as southwest of the Arabian Peninsula, khat has anorexic and euphoric properties. 

The next section of the Special Issue is devoted to dietary supplements. García-Cortés et al. [11] present a tabular listing and clinical characteristics of hepatotoxicity by dietary supplements, which, as products, lack a standard nomenclature or classification scheme and hence are variably defined. Dietary supplements are commonly used as an aid to improve nutritional status, to lose weight, or to treat constipation. They may also be defined as any product intended to supplement but not to substitute the diet. Diagnosing liver injury by dietary supplements is challenging due to the fact that these products are not regulated in the same way as prescription drugs are, and subsequently lack uniform criteria for manufacturing and authentication. Moreover, as occurs with DILI, diagnosis and causality assessment of liver injury by dietary supplements is considered difficult given the absence of diagnostic tests. Attribution of causality is performed through an exhaustive interview with the patients, asking for the chronology of the intake of drugs and herbal and dietary supplements, and the exclusion of alternative causes. The authors also focus on new developments of hepatotoxic concerns originating from illicit androgenic anabolic steroids (AAS) for bodybuilding, as they are frequently sold under the denomination of dietary supplements despite being conventional drugs. AAS are synthetic derivatives of testosterone whose medical indications are mainly male hypogonadism, breast cancer, anemia, and hereditary angioneurotic edema. However, several AAS such as stanozolol, methyltestosterone, oxandrolone, fluoxymesterone, and danazol are also used without medical supervision for performance enhancement and muscle building purposes due to their anabolic effects. The number of AAS hepatotoxicity cases submitted to the Spanish DILI Registry has increased in recent years from 5 cases during the first 15 years (1994–2009) to 15 over the next four years (2010–2013). The reason for this increase could be a combination of increased usage and improved clinical awareness of this form of hepatotoxicity.

Challenges that originated in Hawaii and occurred in connection with 8 suspected clinical cases of liver injury from the use of a dietary supplement (DS) are discussed and critically evaluated by Teschke et al. [12]. Their re-assessment of published cases from Honolulu revealed a lack of causality using RUCAM and considering clinical evaluation. As more than 50% of the US adults consume one or more dietary supplements, it was pointed out that half of the liver patients, who attended a liver center such as the one in Honolulu, will have had a history of DS use, which may lead to the erroneous assumption that the consumed DS is the cause of the liver disease. In fact, a temporal association of DS use and liver disease is not sufficient to claim any causal association. Case record re-assessment revealed numerous confounding variables because some patients used multiple DS and drugs concomitantly, and some patients appeared to have suffered from multiple liver diseases: liver cirrhosis, liver failure by acetaminophen, hepatotoxicity by non-steroidal antiinflammatory drugs (NSAIDs), resolving acute viral hepatitis by hepatitis B virus (HBV), herpes simplex virus (HSV), and varicella zoster virus (VZV), and suspected HEV. Failing to exclude these confounders and to consider more viable diagnoses, specific treatment options may have been missed in some patients. There is also the note that regulatory epidemiologists had reviewed the clinical records of all Honolulu cases for diagnosis correctness and data consistency, which is difficult to reconcile in view of the numerous inconsistencies that had been published. Whenever liver injury cases are published in peer-reviewed medical journals, the scientific community expects that clinical case assessment is in line with standards of mainstream medicine, all relevant and not only selective case data being presented, and alternative diagnoses being appropriately excluded. This refers also to HEV, which easily masquerades as a DILI or HILI. Furthermore, unjustifiable RUCAM score upgrading invalidates case assessment.

Finally, but certainly not of least importance, Avigan et al. [13] cover the broad field of dietary supplements and outline some regulatory perspectives in liver injury due to herbal and dietary supplements in the US, where the risk of hepatotoxicity linked to the widespread use of certain herbal products has gained increased attention among regulatory scientists. Based on current US law, all dietary supplements sold domestically, including botanical supplements, are regulated by the Food and Drug Administration (FDA) as a special category of foods. Under this designation, regulatory scientists do not routinely evaluate the efficacy of these products prior to their marketing, despite the content variability and phytochemical complexity that often characterizes them. Nonetheless, there has been notable progress in the development of advanced scientific methods to qualitatively and quantitatively measure ingredients and screen for contaminants and adulterants in botanical products when hepatotoxicity is reported. In fact, the large number of available dietary supplements is both impressing and disturbing, considering that, in the United States alone, more than 50.000 dietary supplements were marketed between 1995 and 2015. This large number of products seems difficult to be handled in the regulatory context and calls for some product restriction and more regulatory surveillance. Preference should be given to products that are to be used as medicines and fulfill global regulatory requirements of efficacy and safety. Limited details are presented on regulatory approaches to how diagnoses of clinically suspected cases of liver injury by dietary supplements are ascertained. In particular, a diagnostic protocol which would allow comprehensive exclusion of competing diagnoses, such as hepatitis A, B, C, and E, to name just a few possible alternative diagnoses, is missing. It is also unclear how incomplete data sets of clinical cases are evaluated and considered as variables that may substantially confound the final diagnoses or even dismiss the initial diagnoses of dietary supplement-induced liver injury.

As guest editors of this Special Issue with overall 12 contributions, we thank all experts who accepted our invitation and devoted their time and energy to write their papers. These publications covered the broad field of liver injury by drugs, herbs, and dietary supplements and also illustrate how crucial an early and correct diagnosis is in order to arrive at the appropriate therapy. It was a pleasure for us to work together with the authors and the academic editor Prof. Dr. Johannes Haybaeck and his competent editorial team to get this exciting job done. They had chosen for each submitted paper at least three experts in the field as external and independent peer-reviewers whose recommendations were highly appreciated and substantially improved the quality of the articles as they were finally accepted for publication.

Thank you again to all who contributed, peer-reviewed, and assisted to get this special issue published.

## Figures and Tables

**Table 1:** Summary of papers in the Special Issue, arranged by topic as pertaining to liver injury.

Authors	Title	Topics/Keywords	Type
Danan et al. [2]	RUCAM in Drug and Herb Induced Liver Injury: The update	Causality Assessment; Drug and Herb Induced Liver Injury	Review
Hayashi [3]	Drug-Induced Liver Injury Network Causality Assessment: Criteria and Experience in the Unites States	Hepatotoxicity; Causality; Diagnosis; Expert Opinion	Review
Björnsson [4]	Hepatotoxicity by Drugs: The Most Common Implicating Agents	Hepatotoxicity; Drugs; Drug-Induced Liver Injury; Idiosyncrasy	Review
Ortega-Alonso et al. [5]	Case Characterization, Clinical Features and Risk Factors in Drug-Induced Liver Injury	Risk Factors; DILI	Review
Bessone et al. [6]	The Latin American DILI Registry Experience: A Successful Ongoing Collaborative Strategic Initiative	Hepatotoxicity; Drug-Induced Liver Injury Spanish DILI Registry; Latin-American DILI Registry	Review
Frenzel et al. [7]	Herbal Hepatotoxicity: Clinical Characteristics and Listing Compilation	Hepatotoxicity; Herbal Drug; Herb Induced Liver Injury; (HILI); Herbal Hepatotoxicity	Review
Valdivia-Correa et al. [8]	Herbal Medicine in Mexico: A cause of Hepatotoxicity. A Critical Review	Herbal Medicine; Herb-Induced Liver Injury (HILI); Hepatotoxicity; Herbal Dietary Supplements (HDS); Adverse Events; Regulation	Review
Douros et al. [9]	Herb-Induced Liver Injury in the Berlin Case-Control Surveillance Study	Hepatotoxicity; Phytotherapeutics; Pharmacovigilance	Article
Pantano et al. [10]	Hepatotoxicity Induced by “the 3Ks”: Kava, Kratom, and Khat	Kava; Khat; Kratom; Hepatotoxicity; Herbals; Herb Induced Liver Injury	Review
García-Cortés et al. [11]	Hepatotoxicity by Dietary Supplements: A Tabular Listing and Clinical Characteristics	Dietary Supplements; Liver Injury; Anabolic Steroids; Green Tea; Herbalife Products; Hydroxycut; OxyELITE Pro; Vitamin A; Usnic Acid	Review
Teschke et al. [12]	The Honolulu Liver Disease Cluster at the Medical Center: Its Mysteries and Challenges	Herbal dietary Supplements; Centers for Disease Control and Prevention; Food and Drug Administration; Hawaii Department of Health; OxyELITE Pro; Acetaminophen	Review
Avigan et al. [13]	Scientific and Regulatory Perspectives in Herbal and Dietary Supplement Associated Hepatotoxicity in the United States	US Food and Drug Administration; Regulation; Dietary Supplement Health and Education Act (DSHEA); Herbal Supplement Epidemiology; Herbal Supplement Contamination; Herbal Supplement Adulteration	Review
